# Functional Importance of Mobile Ribosomal Proteins

**DOI:** 10.1155/2015/539238

**Published:** 2015-09-20

**Authors:** Kai-Chun Chang, Jin-Der Wen, Lee-Wei Yang

**Affiliations:** ^1^Institute of Molecular and Cellular Biology, National Taiwan University, Taipei 10617, Taiwan; ^2^Institute of Bioinformatics and Structural Biology, National Tsing Hua University, Hsinchu 30013, Taiwan

## Abstract

Although the dynamic motions and peptidyl transferase activity seem to be embedded in the rRNAs, the ribosome contains more than 50 ribosomal proteins (r-proteins), whose functions remain largely elusive. Also, the precise forms of some of these r-proteins, as being part of the ribosome, are not structurally solved due to their high flexibility, which hinders the efforts in their functional elucidation. Owing to recent advances in cryo-electron microscopy, single-molecule techniques, and theoretical modeling, much has been learned about the dynamics of these r-proteins. Surprisingly, allosteric regulations have been found in between spatially separated components as distant as those in the opposite sides of the ribosome. Here, we focus on the functional roles and intricate regulations of the mobile L1 and L12 stalks and L9 and S1 proteins. Conformational flexibility also enables versatile functions for r-proteins beyond translation. The arrangement of r-proteins may be under evolutionary pressure that fine-tunes mass distributions for optimal structural dynamics and catalytic activity of the ribosome.

## 1. Introduction

Translation of the genetic code into functional proteins is carried out by the ubiquitous ribosome throughout all domains of life. In bacteria, the 50S large subunit and the 30S small subunit assemble into the 70S ribosome after translation initiation. The 30S subunit is composed of 16S rRNA and about 20 proteins, while the 50S subunit contains 23S rRNA, 5S rRNA, and more than 30 proteins [1]. Some of the major activities of the ribosome come from rRNAs, which take up two-thirds of the molecular weight of the 70S ribosome. In fact, ribosome ratcheting, which is the largest global conformational change of the ribosome during translocation, is shown to be encoded in the topology of the rRNAs according to elastic network modeling (ENM) [2]. The 16S rRNA plays major roles in recognition of the initiation Shine-Dalgarno (SD) sequence and selection of the A-site tRNA [3], while the 23S rRNA harbors the peptidyl transferase center (PTC, Figure 6), which is stabilized by Mg^2+^ ions [4, 5] and catalyzes peptide bond formation without the aid of ribosomal proteins (r-proteins) [6, 7]. Interestingly, before the major oxygenation events caused by photosynthetic species, Earth was abundant in soluble iron (Fe^2+^) [8], which may serve as an important RNA cofactor, rather than Mg^2+^. In an anoxic solution that contains Fe^2+^ instead of Mg^2+^, 23S rRNA is found to catalyze electron transfer in a standard peroxidase assay [9]. These observations support the notion that the ribosome is a ribozyme originating from the putative RNA world [7, 10] that nourishes the earliest life forms on earth.

What functions, then, do r-proteins contribute to during translation? Many r-proteins are essential for cell survival, as in the case of S4 protein that not only guides early 16S rRNA folding and 30S subunit assembly [11–13] but also unwinds mRNA structures during translation [14].* Escherichia coli* mutants lacking other r-proteins, including L1, L11, L33, S6, and S9 proteins, remain viable [13, 15, 16]. Therefore, these r-proteins have been chosen as fluorescence labeling sites in single-molecule Förster resonance energy transfer (smFRET) experiments that unravel the translational dynamics of the ribosome [17, 18]. The L12 protein is a special case where it is the only r-protein to exist as multiple copies of dimers on the ribosome [19], but only one dimer is required for the cell to survive, albeit at a lower growth rate [20]. The L9 protein is even more intriguing where despite its conservation throughout bacteria, the L9-deletion* E. coli* mutant does not show substantial defects in cell growth [21, 22]. L9 is absent in archaeal and eukaryotic ribosomes [23], indicating that it is actually dispensable for most of the translational activities. In fact, 22 out of 54 r-proteins are shown to be nonessential when deleted individually in* E. coli* [24]. This raises several curious questions including why these proteins are preserved during the course of evolution and whether their absence directly impacts the conformational dynamics of the ribosome or they associate with cellular functions through more indirect ways.

**Figure 1:**
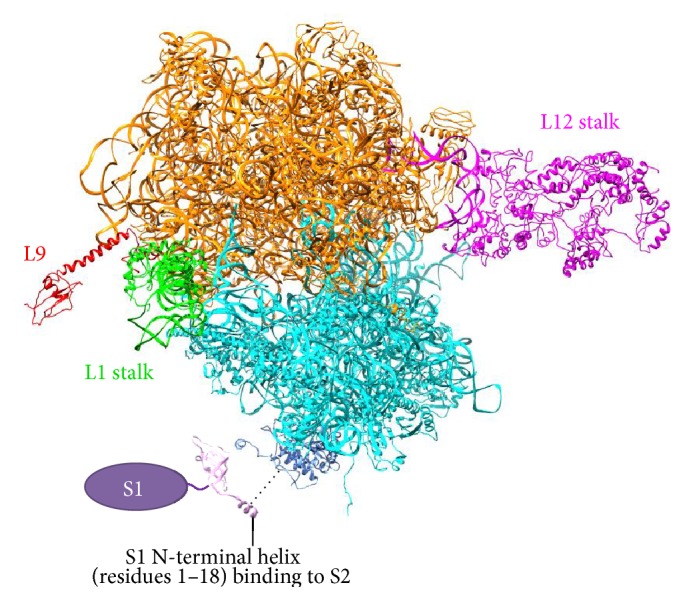
Ribosomal components discussed in this paper. Modeling of the ribosome is described in Methods. Orange: 50S subunit. Cyan: 30S subunit. Red: L9 protein. Green: L1 stalk, composed of the L1 protein and H76–78 (nucleotide 2093–2196) of 23S rRNA. Magenta: L12 stalk, including nucleotide 1030–1124 of 23S rRNA and r-proteins of L10, L11, and L12. Details of L12 stalk are depicted in Figure 4. Due to the flexibility of S1, only a fragment of the S1 NTD in complex with S2 is resolved by X-ray (PDB 4TOI [29]), and the rest of S1 is represented by oval. Here, the S1 protein is not included in the ribosome model, and the interaction between S1 N-terminal helix and S2 is indicated by dashed line.

Structures of most of the r-proteins have been well resolved in 70S ribosome complexes, with some exceptions including the S1 protein and the L1 and L12 stalks which are highly mobile and often missing in X-ray-solved structures. Despite the growing repertoire of ribosome structures from different species with novel techniques, structures of the r-proteins L12 and S1 have never been fully determined in complex with the ribosome. Interestingly, S1 not only has high conformational flexibility [25, 26] but also associates weakly with the ribosome [27]. The unstructured N-terminal domain (NTD) of S1 folds upon binding to the ribosome in a way similar to many intrinsically disordered proteins (IDPs) [28, 29]. Since there has been an increasing interest in the folding and functionality of IDPs, and since the ribosome also exploits the conformational flexibility of some r-proteins for factor recruitment and modulation of protein synthesis, here we review some of the bacterial r-proteins, namely, S1, L9, L1 stalk, and L12 stalk (Figure 1), which lack structural information and may function through their intrinsic flexibility.

## 2. S1 Protein Does Not Always Stay on the Ribosome but Participates in Various Functions Other than Translation

Structurally, the S1 protein contains six repeated domains (D1–D6) with flexible linkers in between, and each of the domains is made of the oligonucleotide-binding (OB) fold. The N-terminal flexible segment (residue Met1 to Thr18) is disordered in its free form, while the first 11 residues fold into an *α*-helix upon binding to the S2 protein in the 30S subunit [29] (Figure 1). The interaction seems to be weak and reversible [27]. On the other hand, the OB folds of S1 bind stably to the single-stranded form of RNA during thermal breathing [36]. Thus, S1 acts a passive mRNA helicase [37] that is important for the ribosome to initiate translation on an mRNA with a structured 5′ untranslated region (5′ UTR) [29, 38].

In addition to unfolding and delivery of an mRNA to the ribosome, S1 participates in an array of cellular functions. Together with elongation factors EF-Tu and EF-Ts in the* E. coli* host, as well as the phage-encoded *β*-subunit, S1 is one of the four subunits of the Q*β* phage RNA replicase holoenzyme [39, 40]. It also associates with the *β* protein from *λ* phage to form a component of general recombination [41]. It promotes enzymatic activities including transcriptional cycling* in vitro* [42] and RNA-cleavage by the T4 phage endoribonuclease RegB [43]. The versatility of S1 seems to be enabled by multiple OB folds strung together in a way that can interact with different RNAs. Even the unstructured N-terminus plays an important role of binding to the ribosome. Unlike most of the ribosomal proteins, the acidic residue composition of S1 prevents tight binding with the rRNA scaffold of the ribosome [38, 44]. Reversible association may be beneficial for the S1 protein to cycle between different cellular components, especially between various mRNAs with structured 5′ UTR.

## 3. Allosteric Regulation of L1 Stalk Controls tRNA Translocation and Dissociation

**Figure 2:**
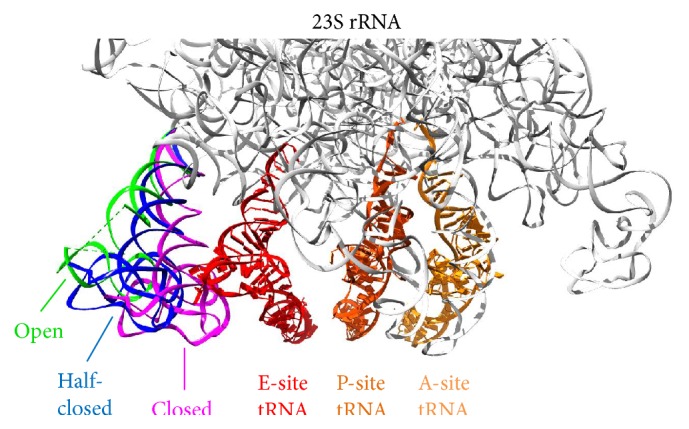
Different conformations of L1 stalk. The L1 protein is not resolved in these structures, and therefore, only the H76-78 of 23S rRNA (nucleotide 2093–2196) part of the stalk is shown and highlighted. An open conformation (green, PDB 2I2T [30]) is found in the ribosome lacking an E-site tRNA (red). When the tRNAs are in their A/P and P/E hybrid states, L1 stalk adopts a fully closed conformation (magenta, PDB 3R8S [31]). In the presence of a classical-state E-site tRNA, L1 stalk resides in between those conformations and thus becomes “half-closed” (blue, PDB 3I8I [32]). The A-site (yellow), P-site (orange), and E-site (red) tRNAs in their classical states and the 23S rRNA (gray) are taken from PDB 3I8I [32].

The entire L1 stalk is comprised of the L1 protein and helices 76–78 (denoted as H76–78 herein) of domain V of the 23S rRNA. For the L1 protein, there are two domains separated by a conserved and flexible hinge region made of Gly67, Gly69, and Gly159. Domain I includes residues 1–67 and 160–234, while Domain II includes residues 68–159. Despite the well-defined structure of L1 protein solved in isolation [45], it is missing in many ribosome structures due to the high mobility of the entire L1 stalk, and at least three major configurations have been observed from structural and single-molecule studies [3, 17] (Figure 2).

After peptide bond formation, the 30S rotates 4–12° counterclockwise relative to the 50S when viewed from the solvent side of 30S (termed “rotated state”) [46, 47]. Ribosome translocation is then facilitated by fluctuation of tRNAs between the classical A/A and P/P states and the hybrid A/P and P/E states (the letter before slash denotes the site where the anticodon-stem loop (ASL) binds in 30S, while the other letter is the site where the acceptor stem binds in 50S) [48]. The mobility of the L1 stalk may direct the movement of a deacylated tRNA from P/E state to E/E state [49, 50], where the tRNA dissociates spontaneously [51]. Previous structural studies showed that when the E-site is vacant (nonrotated ribosome), the L1 stalk adopts an open conformation (e.g., PDB 2I2T [30]). When a P/E-state tRNA is present (pretranslocational rotated ribosome), the stalk is fully closed to interact with the tRNA (e.g., PDB 3R8S [31]). A half-closed stalk is observed when an E/E-state tRNA is present in a posttranslocational nonrotated ribosome (e.g., PDB 3I8I [32]) (Figure 2). Single-molecule FRET experiments further revealed that the L1 stalk fluctuates between open and closed configurations in the pretranslocational ribosome, and that L1 stalk opening is strongly suppressed after binding of EF-G [52].

**Figure 3:**
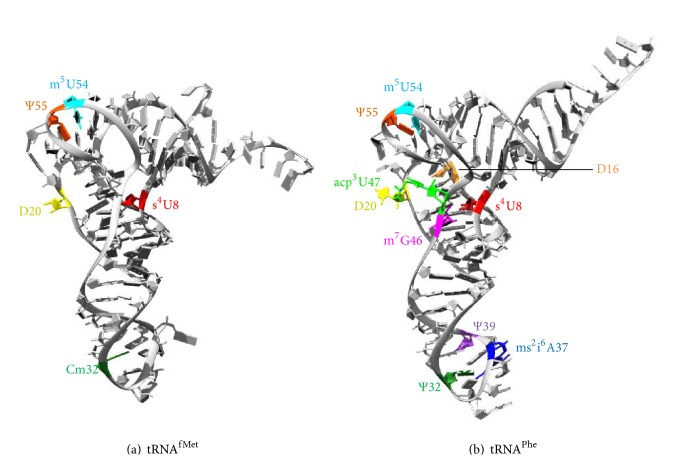
Natural RNA modifications [33] in tRNA^fMet^ ((a) PDB 2FMT [34]) and tRNA^Phe^ ((b) PDB 3IZW [35]). ms^2^i^6^A = 2-methylthio-*N* ^6^-isopentenyladenosine; m^7^G = 7-methylguanosine; D = dihydrouridine; Ψ = pseudouridine; m^5^U = 5-methyluridine; s^4^U = 4-thiouridine; Cm = 2′-*O*-methylcytidine; acp^3^U = 3-(3-amino-3-carboxypropyl)uridine.

The L1 protein has several basic residues to form salt bridges with the acidic tRNA backbone in P/E state, but it forms less salt bridges with the initiator tRNA^fMet^ than with the elongator tRNA^Phe^. The L1 stalk rRNA also has a weaker stacking interaction with P/E-state tRNA^fMet^ [49]. Taken together, the L1 stalk has a lower affinity for tRNA^fMet^ as compared to tRNA^Phe^, and it opens more frequently in the presence of a P/E-state tRNA^fMet^ [52]. Interestingly, when all modifications of the ribonucleosides in P/E-state tRNA^fMet^ (Figure 3(a)) are excluded during molecular dynamics (MD) simulations, Domain II of L1 protein seems to become very flexible and move independently of Domain I [49]. Therefore, both the identity and the chemical constituents of P/E-state tRNA affect the movement of L1 stalk. The lower affinity with L1 stalk and the resulting slower translocation kinetics of initiator tRNA^fMet^ (with properly modified ribonucleosides) may help to stabilize the initiation complex. Modified ribonucleosides are also important for the functions of elongator tRNAs, such as tRNA^Phe^ (Figure 3(b)). They stabilize tRNA folding [53] and modulate tRNA binding with L1 stalk [49].

In summary, the conformational dynamics of L1 stalk is affected by both local properties of P/E-state tRNA and the aforementioned allosteric binding of EF-G to the ribosome. Surprisingly, a smFRET study further found that encounters with downstream mRNA structures decrease tRNA dissociation rate without affecting tRNA translocation rate [54]. Since both tRNA translocation and dissociation are modulated by L1 stalk, the results indicate a long-range communication between L1 and the mRNA entrance.

## 4. Mobility of L12 CTD Is Regulated to Ensure Proper Delivery of Translational GTPases

**Figure 4:**
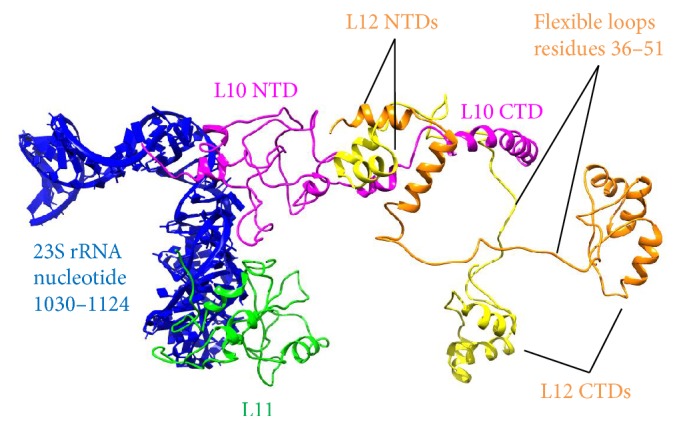
Components of L12 stalk. See Methods for the modeling details. Blue: 23S rRNA nucleotide 1030–1124. Magenta: L10. Green: L11. Orange and yellow: a pair of L12 dimers. For clarity, only one L12 dimer is shown. L12 monomers dimerize through their NTD helices, which also bind to L10 CTD. L10 CTD accommodates two (in* E. coli*) to three (in* T. thermophilus*) L12 dimers [19]. L10 NTD attaches to 23S rRNA.

Multiple copies of the L12 homodimer bind to the L10 C-terminal domain (CTD) to form a protein complex. Notably, L12 and S1 are the only r-proteins known to associate with other r-proteins, instead of with rRNAs. L10 (in complex with L12 dimers) and L11 directly associate with a region of 23S rRNA (nucleotides 1030–1124) to form the L12 stalk (Figure 4). The L12 protein is also referred to as L7/L12 protein. L7 and L12 are identical proteins, except that L7 is posttranslationally acetylated at the N-terminus in* E. coli*. Although the structure of L12 homodimer has been determined in isolation by nuclear magnetic resonance (NMR) [55], the complex formed by L10 CTD and L12 dimers, being the most mobile region of the L12 stalk, has never been seen in the ribosome complex. The L12 protein contains a V-shaped NTD and a globular CTD joined by a flexible loop (residues 36–51). Each monomer of L12 NTD is formed by two antiparallel helices and serves as a dimerization module to interact with the NTD of another L12 monomer (Figure 4). The L10 CTD is capable of accommodating multiple copies (two for* E. coli* and three for* Thermus thermophilus*) of L12 NTDs, and the number of copies is dictated by the length of L10 CTD. A minimum of two copies of L12 dimers is found in* E. coli*, while up to four copies have been found in the cyanobacteria* Arthrospira platensis* [19]. It remains a mystery why the ribosome requires multiple copies of L12 dimers (equivalent to P1/P2 dimers in eukaryotes [56]) to achieve optimal initiation and elongation efficiency [20].

The L12 CTD is responsible for interacting with translation factors [57]. Since L12 CTDs are highly mobile and exist as multiple copies of dimers, L12 CTDs have been proposed to recruit and deliver elongation factors EF-Tu and EF-G to the ribosomal factor binding site by increasing the encounter frequency, and thereby leading to association rates higher than expected for random collisions [57].

After initial encounter with a translation factor, such as IF2, EF-Tu, EF-G, or RF3, the L12 CTD may facilitate loading of the translation factor into the factor binding site jointly with L11's NTD through a conserved “proline switch” mechanism [58]. It has been demonstrated that EF-G can drive* cis-trans* isomerization of the proline switch (PS22) on L11 through the peptidyl-prolyl* cis-trans* isomerase (PPIase) center located between the G-domain and Domain V of EF-G. The* cis* form of PS22 enables the L11 NTD to interact and immobilize the L12 CTD, and thereby allowing full accommodation and subsequent GTP hydrolysis of EF-G. PS22 is then “switched off” to the* trans* form possibly by GDP-bound EF-G through an unknown mechanism [58]. Thus, the EF-G, functioning as a GTPase, PPIase, and a translocase that promotes translocation of the translation complex, facilitates its own binding to the ribosome by indirectly altering the mobility of the L12 CTD. In contrast, when the intrinsic mobility of L12 CTD is restricted by shortening the flexible loop between NTD and CTD, the translation activity is comparable to that of L12-depleted ribosomes, but doubling the length of linker has limited effects [59, 60]. The proline switch mechanism may be universally conserved for other translational GTPases in all three domains of life [58].

## 5. Bacterial L9 Protein Is Conserved and yet Nonessential for Translation

The bilobed architecture of L9 protein consists of a globular NTD docking into 23S rRNA, a long helix linker, and a globular CTD [61] (Figure 1). In all crystal structures of the wild-type ribosome, L9 extends its CTD far away from the ribosomal surface and contacts with the 30S subunit of a neighboring ribosome. Depletion of L9 leads to different crystal forms, which allows resolving ribosomes in complex with translational GTPases [62]. Indeed, L9 adopts a distinct bent conformation toward the S6 protein in a cryo-electron microscopy (cryo-EM) structure [63]. Notably, although elongation factors are occluded by the neighboring ribosome's L9 in crystal packing, both open (seen in X-ray structures) and bent (seen in the recent cryo-EM structure) conformations of L9 do not actually clash with nearby elongation factors in the organization of polysomes [63, 64]. Whether L9 coordinates polysome formation by bridging neighboring ribosomes remains unknown.

The functional role of L9 in reading frame maintenance is most discernible during expression of T4 phage* gene60*. The gene contains a bypass region where the ribosome recognizes the nascent peptide signal and the mRNA hairpin and then “hops” a 50-nucleotide gap before resuming translation [65, 66]. The* hop-1* mutation, which is a Ser93Phe alteration in the L9 CTD, is found to partially restore bypassing efficiency in the absence of a stable* gene60* hairpin. Interestingly,* hop-1* mutation does not increase backward frameshifting efficiency, but complete depletion of L9 increases both forward slippage and backward slippage. Therefore, L9 is proposed to block backward slippage by posing a steric hindrance between neighboring ribosomes, while forward slippage may be suppressed by specific interactions between the L9 CTD and the upstream neighboring ribosome [67].

In addition to the phage-specific gene, massive occurrence of programmed translational bypassing elements (byps) is found in mitochondria [68]. These byps may originate from intron-like mobile genetic elements [69]. Subsequently, phages may contribute to evolutionary diversification of bacteria by propagating these mobile byps. Considering the extensive coevolution of bacteria and phages [70], and the possible bacterial origin of mitochondria, their ribosomes may evolve to ensure proper translation of genes bearing byps. Interestingly, no L9 homolog has been found in eukaryotic 80S ribosome [23, 71]. This may be due to the facts that L9 has more prominent functions during translation of phage-specific byps and that eukaryotic cells are much less dependent on virus for diversification of gene pools.

Similar to S1, L9 may participate in cellular processes other than translation. Although L9 deletion mutants do not exhibit appreciable growth phenotypes, mutations in the essential ribosome biogenesis GTPase Der protein cause dependence on L9. For the Thr57Ile mutation, which impairs the GTPase activity of Der, L9 depletion leads to an aberrant, elongated cell morphology and a defect in cell division. Interestingly, L9 does not rescue the GTPase activity of Der* in vitro*, suggesting that L9 may not directly interact with Der to complement the defective phenotypes. Since L9 NTD, which binds to 23S rRNA, is sufficient to complement the* der* mutant, L9 may share a similar function with Der in promoting and/or stabilizing correct assembly of the 70S ribosome [21]. However, the precise physiological functions of L9 and Der remain to be uncovered.

## 6. A Mechanical View of the Ribosome and r-Proteins

**Figure 5:**
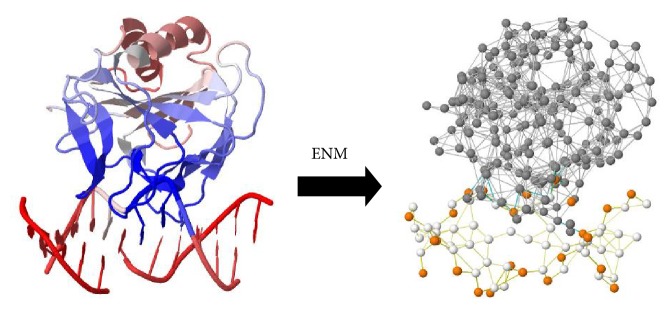
Coarse-graining of demethylase AlkB (PDB 4NIH) in ENM. For each amino acid, the C_*α*_ atom is taken as the representative node. Three nodes, namely, the P atom of the phosphate group, the C2 atom of the nitrogenous base, and the C4′ atom of the pentose, are chosen to represent a nucleotide. The difference in the number of nodes reflects the fact that the average molecular weight of each amino acid is ~110 Da, while that of each nucleotide is ~330 Da. Here the simple harmonic potentials between nodes within a cut-off distance of 15 Å are denoted by lines.

Intrinsic dynamics of a protein are encoded in the topology of its native contacts [72–76]. Elastic network model (ENM), a coarse-grained version [77–79] of normal mode analysis [80, 81] (Figure 5), has been extensively used since the mid-90s to study the intrinsic dynamics of biomolecules [74, 82, 83], especially for supramolecular protein (or protein/nucleic acid) assemblies [84–88]. In ENM, the molecular structure of interest is coarse-grained to the residue level as nodes, with interactions between these nodes being approximated by a simple harmonic potential [77–79]. Taking anisotropic network model (ANM), the most broadly used ENM, as example, the potential E_ANM_ takes the form

(1) $$\begin{array}{c} E_{\text{ANM}}=\frac{\gamma }{2}\left[\;\overset{N}{\underset{i,j}{\sum }}{\left(\;R_{ij}-R_{ij}^{0}\;\right)}^{2}\Theta \left(\;R_{c}-R_{ij}\;\right)\;\right]. \end{array}$$

Here, *γ* is the uniform spring constant and *N* is the number of nodes in the network; *R*_*ij*_ and *R*_*ij*_^0^ are the distances between the *i*th node and the *j*th node at an instantaneous moment and at the equilibrium state (obtained from experimentally solved structures), respectively. The Heaviside step function, Θ, equals 1 for node pairs with separation shorter than a cut-off distance *R*_*c*_ (i.e., *R*_*c*_ − *R*_*ij*_ > 0) and equals zero otherwise. Predicted thermal motions, in the form of a fluctuation matrix (or interchangeably referred as “covariance matrix”) comprising node-node (auto)correlations, can be derived from

(2) $$\begin{array}{c} \langle \;\Delta R\Delta R^{T}\;\rangle =\frac{k_{B}T}{\gamma }H^{-1}=\frac{k_{B}T}{\gamma }\overset{3N}{\underset{k=7}{\sum }}\frac{1}{\lambda_{k}}V_{k}V_{k}^{T}, \end{array}$$

where Δ**R** is a 3*N*-dimensional displacement vector and Δ**R** = (Δ*x*_1_Δ*y*_1_Δ*z*_1_ ⋯ Δ*z*_*N*_)^*T*^ for *N* nodes in 3-dimensional space. *k*_*B*_ is the Boltzmann constant, and *T* is the absolute temperature. **H** is the Hessian, a force constant matrix encoded by protein contact topology at equilibrium [74, 82, 83, 89]. The covariance matrix, derived from the inverse of Hessian, can be further decomposed into the sum of an orthonormal basis set, the normal modes. The resulting *λ*_*k*_ and **V**_*k*_ from eigenvalue decomposition are the *k*th smallest eigenvalue and the corresponding eigenvector, respectively. The first six eigenvalues are equal to zero, corresponding to degrees of freedom for rigid-body rotation and translation in 3-dimensional space. Each **V**_*k*_ is a form of vibrational motion that the biomolecule can perform (the *k*th normal mode), with its frequency being the square root of *λ*_*k*_.

ENM has been applied to refine structures [90, 91] and extract residue-level information from electron paramagnetic resonance (EPR) [92], smFRET [93], and cryo-EM [94]. It is also indispensable to study supramolecules' dynamics such as the ribosome. Molecular dynamics (MD) simulations [95], a powerful chemical technique that was developed since the 50s and enjoyed a delayed acknowledgement with Nobel Prize awarded in 2013, have provided descriptions of ribosomal dynamics up to a couple of hundred nanoseconds [96, 97]. The time scale is however a few order of magnitudes shorter than, say, the well-known ratcheting motion of the ribosome that is characterized experimentally by X-ray [98] and cryo-EM [46] and known to occur on the timescale of milliseconds to seconds. On the other hand, ENM was shown to well capture such motion in a few studies [2, 87, 99]. It therefore confirms that ratcheting motion, a relative rotation between 30S and 50S subunits, is intrinsic at room temperature and encoded mainly by rRNAs' contact topology [2]. Furthermore, residues at the mRNA entrance of the ribosome which exhibit correlated motions with the mRNA were readily revealed by ENM and were proposed to be the active sites of the ribosomal helicase [2], some of which have already been supported experimentally [14]. It is noteworthy that protein dynamics predicted by ENM generally locate known catalytic residues and docking interfaces around vibrationally and rotationally inert regions, making efficient predictions of functional sites possible [75, 89, 100]. This tendency can be explained by the fact that a preorganized and rigid catalytic site is required to provide stabilizing environment for the transition state of the substrate [101–103].

**Figure 6:**
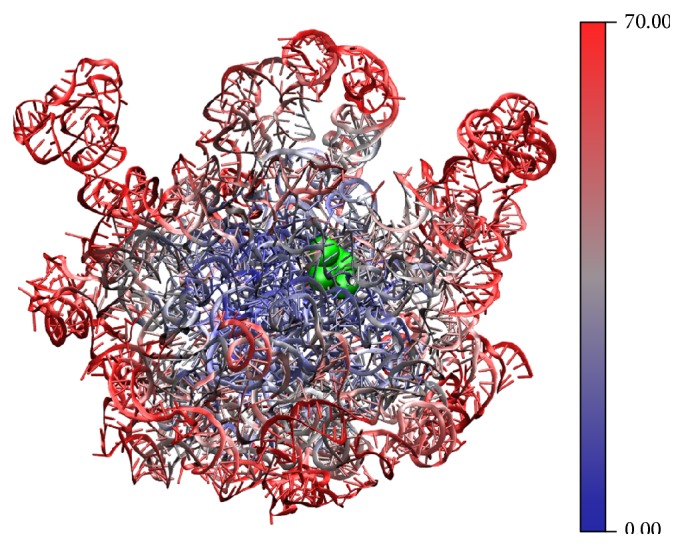
*T. thermophilus* 23S rRNA color coded according to B-factors from 0 Å^2^ to 70 Å^2^ [32]. For visual contrast and clarity, B-factors larger than 70 Å^2^ are all colored red. Nucleotides of PTC, including A2451, U2506, U2585, C2452, and A2602, are shown as green spheres.

Given that high-resolution crystal structures of the ribosome are already available in great detail, the magnitude of thermal motion for each residue can be straightforwardly obtained from experimental B-factors. As expected, the peptidyl transferase center (PTC) is buried in a rigid region of 23S rRNA with low B-factors (Figure 6). Before peptide bond formation, the ribosome is “locked” in the nonrotated state to promote catalysis [104, 105]. Also, PTC may be located around the rotational axis of the 50S subunit, so that the thermally driven rotational motion between 30S and 50S subunits is further minimized. Since the rigid-body rotational axis of an object is determined by its center of mass (CM), we next ask whether PTC lies in proximity to the CM of 50S subunit (CM_50S_). As described in Methods, here we model all missing residues and subunits in the* T. thermophilus* 70S ribosome (PDB 4V6F) [32] by homology modeling (Figure 1). As shown in Figure 6, the PTC, which consists of A2451, U2506, U2585, C2452, and A2602 of 23S rRNA [106, 107], is in proximity to the calculated CM_50S_ (red sphere). Considering the fact that many r-proteins decorate the rRNA core on its periphery without essential functions, it could be that the r-proteins may fine-tune the mass distribution of the ribosome in order to achieve optimal tRNA translocation and peptide bond formation, as suggested by Wang and Jernigan previously [108]. Here we further elaborate this idea by considering the mass balance between L9 and L12 proteins that lie on the opposite sides of the 50S subunit.

**Figure 7:**
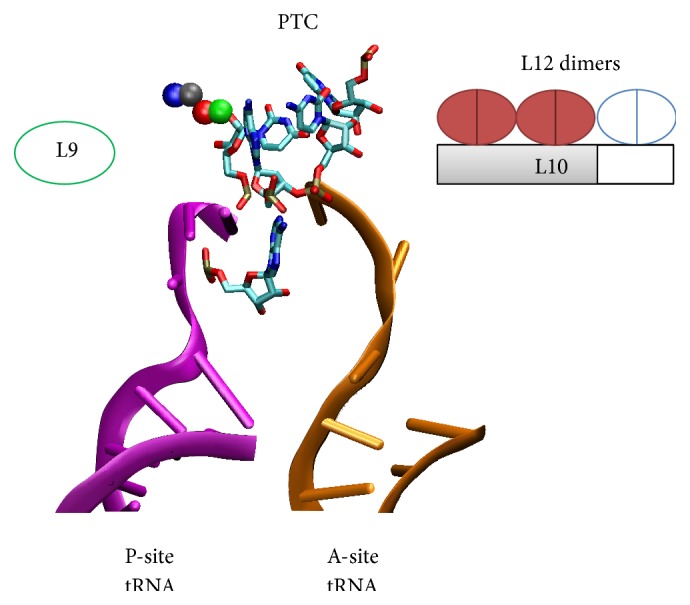
Deletions of r-proteins affect CM_50S_. Calculation of CM_50S_ is explained in Methods. Red sphere: wild-type position of CM_50S_. Blue sphere: mutant's CM_50S_, where the outermost L12 dimer and its bound L10 CTD segment are deleted (right inset). Green sphere: CM_50S_ of the L9 deletion mutant. Gray sphere: CM_50S_ of the compensatory double mutant with aforementioned deletions. Nucleotides of PTC (A2451, U2506, U2585, C2452, and A2602 of 23S rRNA) are shown as sticks.

First, when the outermost L12 dimer is removed along with its binding segment of L10 CTD [19], CM_50S_ tilts towards L9 (Figure 7, blue sphere). On the contrary, when L9 is deleted, CM_50S_ lies closer to the L12 stalk (Figure 7, green sphere). While multiple L12 dimers are required for efficient factor recruitment, L9 may be important for counterbalancing the mass contributed by multiple L12 dimers. In the absence of L9, rotation of the subunits, tRNA translocation, and peptide bond formation may be slightly compromised. This may be the case during translation of the byp in* gene60*, where L9 deletion increases the propensity of ribosome slippage. Consequently, we expect that deleting one copy of L12 dimer should lead to similar phenotype of reduced frame maintenance, which could be partially rescued by removal of L9 (Figure 7, gray sphere). Despite the conserved overall architecture of ribosomes, the rRNA cores from* T. thermophilus* and* E. coli* differ slightly, which may lead to difference in the copy numbers of L12 dimers between the two species. An interesting possibility is that even though increasing the length of L10 CTD, and consequently the number of accommodated L12 dimers, may be advantageous for recruitment and activation of elongation factors [57], the loss of mass balance may compromise translation fidelity and/or speed.

Although L9 and L12 are quite distant from PTC that direct interactions seem unlikely, considering the regulatory roles of L12 dimers in the proper functioning of EF-Tu and EF-G, the possibility remains that they act indirectly through interactions with elongation factors. Given that the ribosome can synthesize oligopeptides without elongations factors and GTP, albeit at a very slow pace [109, 110], one may use such factor-free* in vitro* system to probe how the presence and absence of L9 as well as different copy numbers of L12 dimers regulate translation without interferences from other factors. To further elucidate how the differences in mass distribution may alter local dynamics around PTC, one can apply ENM to study the A-site and P-site tRNAs dynamics for various ribosome mutants lacking or gaining subunits of L9 and L12 dimers. The predictions can then be compared with smFRET experiments, where A-site and P-site tRNAs are fluorescently labeled [111]. The hypothesis that r-proteins (especially L9 and L12 stalk) may act by balancing the mass of the ribosome therefore calls for experimental validations. The mass-balancing arrangements of subunits may be a general scheme for regulating catalytic efficiency in enzymes, which is a desirable feature for rational design of useful enzymes.

## 7. Methods

### 7.1. Modeling the Missing Subunits and Residues of the Ribosome

The elongation complex from* Thermus thermophilus* (PDB 4V6F) served as the starting template for the 70S ribosome model. Missing subunits and residues were modeled by superimposing homologous structures from the PDB database, followed by a 20-step energy minimization with GROMOS 43B1 force field in Swiss PDB-Viewer 4.0.4 [112]. As shown in Figure 1, S1 was not modeled, due to the ambiguity of its structure and binding position and the fact that it associates weakly with the ribosome during elongation. Three L12 dimers were superimposed onto the L10 CTD. Subsequently, L10, L11, and three L12 dimers were subjected to MD simulations with explicit solvent for 5.8 ns to relax the steric clashes. The final result is shown in Figure 1. For the ribosome model with only one L12 dimer, the L10-L11-L12 complex was again equilibrated with MD to produce the final model (Figure 4). Figures were prepared by VMD [113] or UCSF Chimera [114].

### 7.2. Estimating CM_50S_

To represent the overall mass distribution of the ribosome model, three atoms were taken for each nucleotide, namely, P of the backbone, C2 of the base, and C4′ of the pentose. For each amino acid, only C_*α*_ atom was retained, reflecting the difference in average molecular weights of a nucleotide (~330 Da) and an amino acid residue (~110 Da) [115]. Thus, the molecular weight represented by each coarse-grained node was about 110 Da. CM_50S_ was straightforwardly calculated as the geometric center of the constituent nodes.
